# Testing tongue swab samples by Cepheid Xpert MTB/RIF Ultra: comparison of two protocols applied to samples from persons with low-bacillary load TB

**DOI:** 10.1128/spectrum.01493-25

**Published:** 2025-11-12

**Authors:** Rachel C. Wood, Sophie Goodgion, Alaina M. Olson, Angelique K. Luabeya, Mark Hatherill, Gerard A. Cangelosi

**Affiliations:** 1 Department of Environmental & Occupational Health Sciences, University of Washington 7284, Seattle, Washington, USA; 2 South African Tuberculosis Vaccine Initiative, Institute of Infectious Disease & Molecular Medicine and Department of Pathology, University of Cape Town 37716, Cape Town, South Africa; End TB Dx Consulting LLC, San Diego, California, USA

**Keywords:** tongue swab, tuberculosis, Xpert Ultra, sample processing, sensitivity, accuracy

## Abstract

Tongue swabs (TS) are non-sputum specimens for molecular diagnosis of tuberculosis (TB). Analysis of swabs by Cepheid Xpert MTB/RIF Ultra (Xpert Ultra) is less sensitive than the use of certain manual quantitative polymerase chain reaction methods, but is desirable given the widespread use and familiarity of Xpert Ultra. This study evaluated an easy-to-use protocol for analyzing TS by Xpert Ultra. TS samples from symptomatic South African patients were previously collected and tested by Xpert Ultra using a protocol that requires a heating block (termed Heat + TE). Replicate, paired samples from the same patients were tested by a newly reported method that does not require heating (termed 2:1 SR). The diagnostic accuracy of the two methods was compared. This paired comparison prioritized samples from participants with low sputum bacillary loads, a population in whom TS sensitivity is lowest. Eighty-eight were TB-positive by sputum microbiological reference standard (MRS) and 20 were TB-negative (total *N* = 180). Within this sample set, the Heat + TE method was 42.5% (38/88; 95% confidence interval [CI]: 33–54%) sensitive, and the 2:1 SR method was not significantly different at 52.3% (46/88; 95% CI: 41–63%) sensitive relative to sputum MRS. Both methods were 100% (20/20; 95% CI: 83–100%) specific. A secondary analysis compared the sensitivities of single-sample versus dual-sample testing within a cohort (*N* = 241) that included both high- and low-bacillary load participants. Testing of a single sample was 75.5% (182/241; 95% CI: 70-81%) sensitive relative to sputum MRS, whereas dual sampling was 81.3% (196/241; 95% CI: 76–86%) sensitive; the difference was not significant.

**IMPORTANCE:**

Tongue swab (TS) samples are novel, non-invasive, easy-to-collect diagnostic specimens for tuberculosis testing. This study compared two methods for processing TS samples in preparation for analysis using Xpert Ultra, a popular commercial platform for molecular detection of *Mycobacterium tuberculosis* DNA in human specimens. A newly described method has logistical advantages over a previously described method, in that it does not require additional equipment and is less prone to errors. The newly described method was found to be at least as sensitive and specific as the previous method. Therefore, the logistical advantages of the new method come at no cost in terms of diagnostic accuracy, and its adoption is recommended. A secondary analysis indicated that dual-sample testing can modestly boost diagnostic yield relative to testing a single sample.

## INTRODUCTION

Pulmonary tuberculosis (TB) is usually detected by analysis of patient’s sputum, a mixture of saliva and mucus expectorated from the respiratory tract. Sputum collection presents exposure risks to healthcare workers, and many patients cannot routinely produce sputum for testing. Sputum collection can be especially challenging in community settings among minimally or non-symptomatic people who may comprise the majority of TB cases. These challenges have created strong incentives to identify alternative patient samples that are easier to collect from any human, in any setting (1–4).

Some of these needs can be met by tongue swabbing in combination with nucleic acid amplification testing (NAAT) for *Mycobacterium tuberculosis* (MTB) DNA (5–15). Tongue swabbing is fast, painless, and can be done in a multitude of settings. Modeling, user preference, and feasibility studies have indicated that tongue swabbing is preferred by patients and health care providers and has the potential to increase the coverage and yield of TB testing, especially in community settings (16–19).

Much ongoing research has focused on identifying the best readout methods for testing tongue swabs (TS) for MTB DNA. Though not approved by the WHO for widespread clinical use, there is evidence that the most sensitive approaches are NAAT methodologies that were developed explicitly for TS samples (5, 14, 15, 20). In contrast, sputum testing platforms such as Cepheid GeneXpert MTB/RIF Ultra (Xpert Ultra) have exhibited somewhat reduced sensitivity with TS, typically 72–77% relative to sputum microbiological reference standard (MRS) (7, 8, 13). However, Xpert Ultra has the advantage of being widely available in TB clinical settings and familiar to users. Therefore, there is considerable interest in adapting Xpert Ultra to the task of TS testing.

In recent studies, we and others tested TS samples by Xpert Ultra using a sample processing method termed “Heat + TE” (7, 13). The Heat + TE method involves first heating the samples to 100°C for 10 minutes to ensure biosafety, followed by two applications of 1 mL TE Buffer, vortexing to mix, and transferring all the eluate into the Xpert cartridge for a total volume of about 2 mL. A limitation of this method is that the first step requires a heating apparatus, which is not commonly available at many sites that use Xpert Ultra for sputum testing. Additionally, some sites that applied this method to swabs saw pressure error rates as high as 16% (13). More recently, a modified swab processing protocol was reported that does not require heating and instead uses a variation of the Cepheid Sample Reagent (SR) protocol that is routinely used for sputum testing on Xpert Ultra (21, 22). An additional advantage of the new method, termed the “2:1 SR” protocol, is that it is reported to cause fewer overpressure errors on the Xpert Ultra platform than the Heat + TE protocol (21). The 2:1 SR method was reported to have a lower (better) limit of detection than the Heat + TE method in laboratory assessments using contrived samples (21).

Regardless of the analytical method used, numerous studies have shown that people with high bacillary loads in their sputum (e.g., sputum Xpert Ultra semi-quantitative values of high or medium) are consistently positive by TS regardless of the analytical methodology used (5, 7, 8, 14, 15). Therefore, in order to maximize the resolving power of the current comparison of two analytical methods, we focused on participants who had low sputum bacillary loads or were false-negative by the Heat + TE method.

In a secondary analysis, we asked whether the diagnostic yield of TS testing is increased by testing multiple samples from the same individual. During the COVID-19 pandemic, it was common for symptomatic people to exhibit variable COVID-19 test results over the course of their disease (23). We have observed the same with TB oral swabbing. Our first study (12) collected cheek swabs from South African participants with possible TB on three consecutive days. Aggregate sensitivity relative to sputum MRS (swab-positive on at least 1 of the 3 days) was 90% (18/20 participants). However, single-day sensitivities were lower, ranging from 60% to 80% (12/20 to 16/20). Our subsequent study (10) tested two TS samples collected from participants with possible TB over 2 days. Aggregate sensitivity relative to sputum MRS was 83% (49/59) while single-day sensitivities were 71% (42/59) and 78% (46/59) on Day 1 and Day 2, respectively. As with SARS-CoV-2 detection in nasal swabs, MTB pathogen loads in oral swabs may vary from day to day, such that diagnostic yield can be increased by serial sampling and testing. A limitation of past studies on serial TS sampling was the use of manual quantitative polymerase chain reaction methods that are very sensitive but not practical for routine use. Xpert Ultra is more practical for routine use; however, there is a risk that repeat testing with Xpert Ultra will not exhibit the improved diagnostic yield observed with higher-sensitivity methods. Therefore, the current study evaluated the impact of repeat TS testing using Xpert Ultra as the readout.

## MATERIALS AND METHODS

### Study population

This study used banked TS samples (regular Copan FLOQswabs; 520CS01; Copan Italia, Brescia, Italy) that had been previously collected from South African participants. These participants were enrolled and sampled by the University of Cape Town (UCT) and the South African Tuberculosis Vaccine Initiative (SATVI) (7). The University of Washington (UW) Human Subjects Division (STUDY00001840) and the UCT Human Research Ethics Committee (reference number 160/2020) approved this project.

The previous study (7) collected swab samples from two cohorts of volunteers. In Cohort 1, all participants were TB-positive by a sputum MRS. Sputum MRS positivity was defined as a positive result by sputum Xpert Ultra and/or sputum culture. In Cohort 2, participants were recruited and sampled based on symptom criteria and thus comprised both confirmed TB-positive participants and confirmed TB-negative participants. TB positivity in Cohort 2 was confirmed by sputum MRS as in Cohort 1.

### Heat + TE protocol

In the previous study, TS from Cohorts 1 and 2 participants were tested by Xpert Ultra using a method that involved heating the swab samples to 100°C, adding TE buffer, vortexing, and then pipetting the sample solution into an Xpert Ultra cartridge (the “Heat + TE” method) (7). Since multiple samples were collected per participant, there were extra samples from most participants that were archived for use in subsequent studies, including the current one.

### 2:1 SR protocol

The 2:1 SR method used in this analysis was reported by others (21) and proceeded as follows. First, diluted SR was prepared by mixing 8 mL of Cepheid SR buffer and 4 mL of 1× PBS buffer. This solution was vortexed at max speed (Vortex Genie 2, VWR) for 5–10 s and used for same-day testing. Swab samples were removed from the −80°C freezer and thawed on ice for 15 min. Working in a biosafety cabinet, 700 µL of the 2:1 diluted SR was added to each sample. The samples were then vortexed at max speed for 5–10 s, followed by an incubation at room temperature (22°C) for 15 min. Mid-way through the incubation, between 7-8 min, the samples were vortexed again at max speed for 5–10 s. During the incubation, Xpert Ultra cartridges were labeled with the appropriate sample ID and prefilled with 1.5 mL of 1× PBS buffer. After the incubation, the samples were briefly pulse-vortexed, and 500 µL of the sample fluid from the tube containing the swab and 2:1 diluted SR was added to the Xpert Ultra cartridge. The sample fluid was dispensed into the cartridge slowly against the interior wall to avoid generating bubbles. The cartridge was then inserted into the GeneXpert machine and run according to the manufacturer’s instructions.

### Selection of samples for comparison of methods

**Fig 1:**
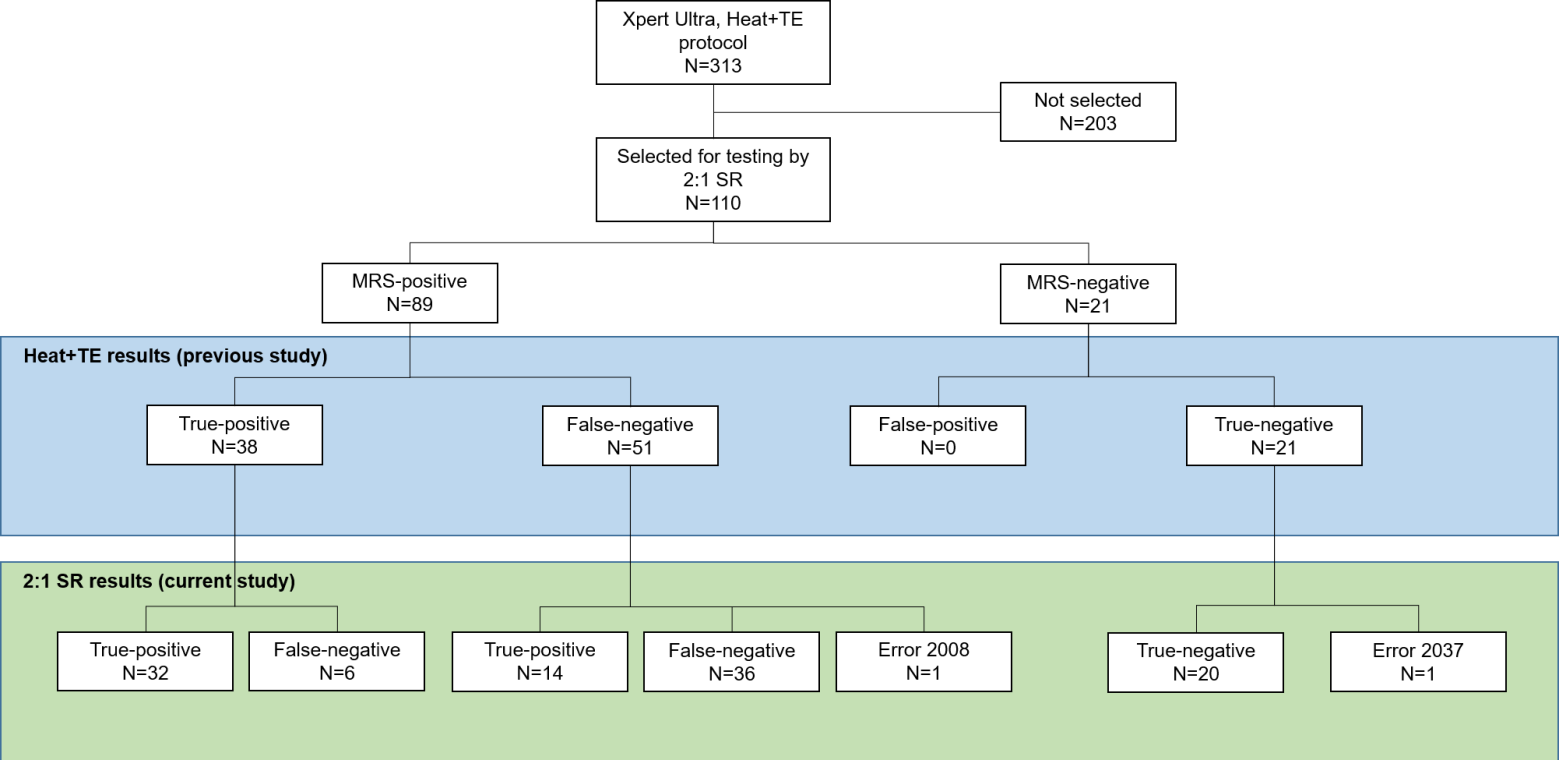
Flowchart displaying the selection of samples tested previously by the Heat + TE method, and the results of testing by the 2:1 SR method.

Sample selection for this analysis is outlined in Fig. 1. Samples were chosen from participants who already had a sample tested by Heat + TE (7). To maximize the rigor and resolving power of the paired comparison between Heat + TE and 2:1 SR, samples from participants with low bacillary loads were prioritized. These were identified in two ways. First, we identified participants (*N* = 38) who were true-positive by Heat + TE in the previous study (7), had low sputum semi-quantitative values by Xpert Ultra testing of sputum, and had banked samples available for testing in the current study. Sputum semi-quantitative values were known only for a subset of Cohort 2 participants; therefore, in the current study, these samples all came from Cohort 2. Of the 38 samples selected for this study, 30 (79%) were from participants with low, very low, or trace values by sputum Xpert Ultra. By comparison, in the original cohort, 32 out of 91 (35%) had sputum semi-quantitative values in these categories (7).

We also tested samples from all participants who were previously sputum MRS-positive and Heat + TE negative (false-negative by TS) (7). We tested all samples from such participants who had a banked sample available for testing (*N* = 26 from Cohort 1, and *N* = 25 from Cohort 2; total *N* = 51).

Because three consecutive TS samples had been collected from each participant in Cohort 1, the three samples were randomized by collection order so that each swab (first, second, or third) was equally represented. In Cohort 2, five Copan TS had been collected on Day 1, and two more had been collected on Day 2. The first two samples from Day 1 and the two from Day 2 were included in the previous study (7). For the current study, any of the five swabs from Day 1 and either of the two from Day 2 were eligible for inclusion. Samples were randomized by collection day and collection order. To do this, each sample was assigned a number and an online random number generator (randomlists.com) was used to make a list of samples.

To assess the specificity of the 2:1 SR method, leftover samples from 21 sputum MRS-negative participants were tested (Fig. 1). These were also randomized by collection day and collection order using the online random number generator.

### Assessment of dual sampling

To assess whether dual sampling boosted aggregate sensitivity, the results of all samples tested by Heat + TE (*N* = 313), as reported in Wood et al. (7) were considered alongside the 2:1 SR results in this study. For each participant who had samples tested by both Heat + TE and 2:1 SR, any positive result by either test was considered a swab positive.

### Statistical analysis

Microsoft Excel and GraphPad Prism were used for statistical analyses. 95% confidence intervals (CIs) were determined using MedCalc Statistical Software that calculates “exact” Clopper-Pearson confidence intervals.

## RESULTS

### Participant characteristics

Replicate swabs from 110 of the 313 participants whose samples were previously tested by Heat + TE (7) were selected for testing by the 2:1 SR protocol as described in Materials and Methods. The sample set included *N* = 89 samples from patients with sputum MRS-confirmed TB, and *N* = 21 samples from randomly selected sputum MRS-negative participants (Fig. 1). The demographic characteristics of these 110 participants are shown in Table 1.

**TABLE 1:** Characteristics of the participants whose samples were tested by the 2:1 SR protocol for Xpert Ultra

	Cohort 1	Cohort 2	
	Sputum MRS-positive (*N* = 26)	Sputum MRS-positive (*N* = 63)	Sputum MRS-negative (*N* = 21)
Age (median, interquartile range)	38 (29–48)	36 (26–47)	37 (28–48)
Gender (male)	15 (57.7%)	35 (54%)	6 (28.6%)
Mixed race ancestry	19 (73.1%)	51 (79.4%)	14 (66.7%)
Black	7 (26.9%)	13 (20.6%)	6 (28.6%)
Asian	0	0	1 (4.8%)
HIV-positive	10 (38.5%)	24 (38.1%)	4 (19%)

### Comparison of Heat + TE to 2:1 SR

Figure 1 summarizes the results. The Heat + TE results in the diagram were generated previously by Wood et al. (7). Testing the samples from 110 participants yielded 108 valid results and 2 Xpert Ultra error results: Error 2008 (pressure error) and Error 2037 (cartridge error).

Among the *N* = 38 participants with true-positive TS results in the previous study, 32 yielded positive results by the 2:1 SR method (Fig. 1). This left six participants whose samples were false-negative by 2:1 SR. Sputum Xpert Ultra semi-quantitative results for those six participants were one medium, three low, one very low, and one unknown. The apparently lower sensitivity of 2:1 SR relative to Heat + TE (84% and 100%, respectively) may have been an artifact of the design of this portion of this study, because all samples were Heat + TE true-positive upon selection. Therefore, we selected a second group of sputum MRS-positive participants who were Heat + TE false-negative (*N* = 51; Fig. 1) and applied the 2:1 SR method to their replicate swab samples. One of these samples yielded an error. Of the remaining 50 swabs, 14 yielded true-positive results by 2:1 diluted SR.

**Fig 2:**
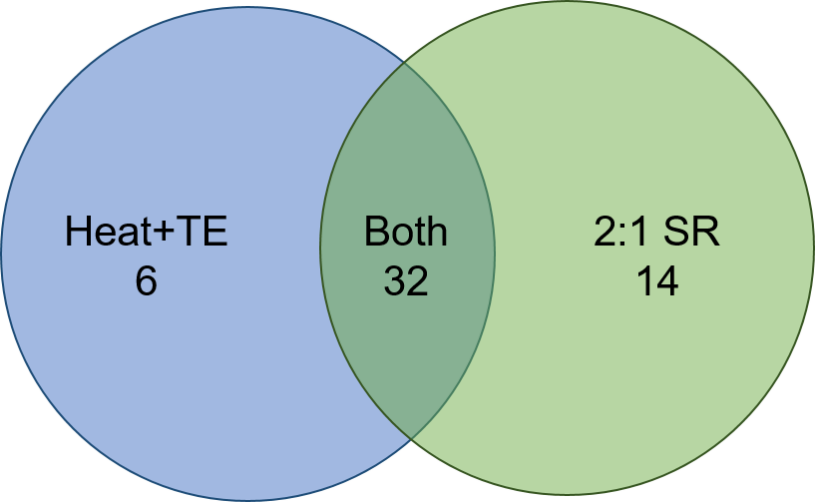
Venn diagram showing the number of true-positive samples by both methods and either method.

Combining the two sputum MRS-positive sample sets (total *N* = 88 excluding the single error), the sensitivities of the two methods are shown in Table 2. The sensitivity of 2:1 SR was higher than the Heat + TE method, but the difference was not statistically significant (*P* = 0.23, two-population proportion *z*-test). Of the 52 true-positives by either method, 32 were positively identified by both Heat + TE and 2:1 SR, while 20 were positively identified by just one or the other method (Fig. 2). Comparing the true-positives to the false-negatives (*N* = 36), there was a significantly higher rate of PLHIV among the false-negatives (58.3%, 21/36) than the true-positives (26.9%, 13/52) (*P* = 0.003, two-population proportion *z*-test). There was no significant difference in rates of PLHIV between the six samples that were Heat + TE true-positive/2:1 SR false-negative and the 14 that were 2:1 SR true-positive/Heat + TE false-negative.

**TABLE 2:** Sensitivity and specificity of the Heat + TE and 2:1 SR protocols relative to sputum MRS

Swab analysis method	Sensitivity (% [*n*/*N*]; 95% CI)	Specificity (% [*n*/*N*]; 95% CI)
Heat + TE	42.5% (38/88); 95% CI: 32.7–54.2%	100% (20/20); 95% CI: 83.2–100.0%
2:1 SR	52.3% (46/88); 95% CI: 41.4–63.0%	100% (20/20); 95% CI: 83.2–100.0%

Sputum Xpert Ultra semi-quantitative results were available for 20 out of 32 participants who were TS true-positive by either method. Participants with low, very low, or trace sputum semi-quantitative results made up 65% (13/20) of the TS true positives, while they made up 100% (9/9) of the TS false-negatives. Sputum Xpert Ultra semi-quantitative results were available for five out of six participants who were 2:1 SR false-negative; three low, one very low, and one medium. Sputum Xpert Ultra semi-quantitative results were available for 4 out of 14 participants who were Heat + TE false-negative; 2 Low and 2 Very Low.

### Impact of dual sample analysis on diagnostic accuracy

Our previous analysis (7) used Heat + TE with Xpert Ultra to test TS samples from 313 South African participants, 241 of whom had sputum MRS-confirmed TB. The sensitivity of the method was 182/241, or 75.5% (95% CI: 69.6–80.8%). The present study tested a second sample from 51 of the 59 participants who were false-negative by TS in the previous study. Fourteen of these samples yielded positive results, albeit by a modified methodology (2:1 SR) relative to the previous study. This raised the combined sensitivity of TS (positive on at least one swab) to 196/241, or 81.3% (85% CI: 75.8–86.0%). The difference in sensitivity between the single sample analysis and the dual sample analysis was not significant (*P* = 0.12, two-population proportion *z*-test).

### TS by Xpert Ultra among people living with HIV

**Fig 3:**
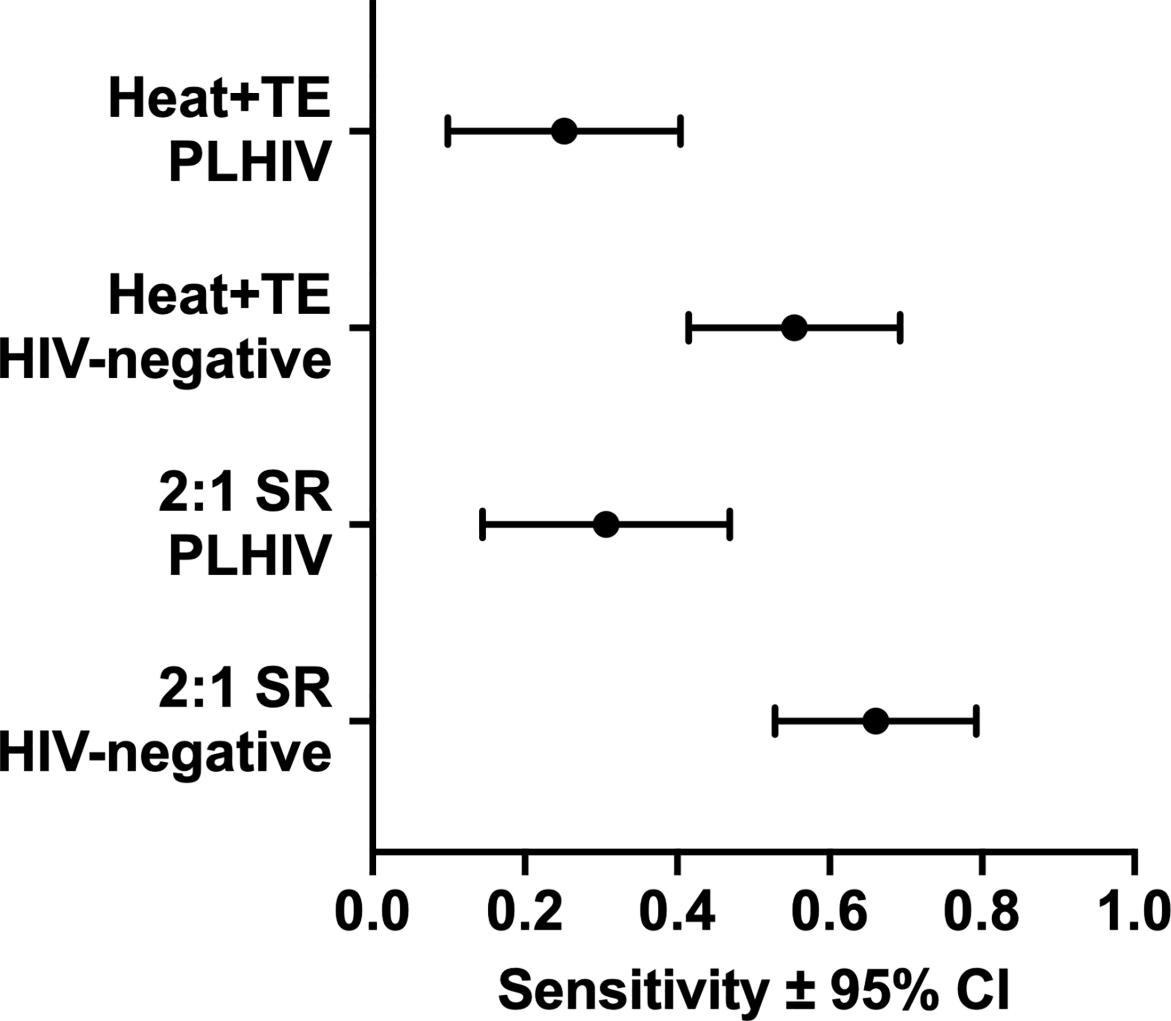
Sensitivities of the two methods relative to HIV status.

Among the entire cohort from which swabs were tested by the Heat + TE protocol (7), 33% (103/313) were PLHIV. Among those participants for whom an extra swab was tested in this study by the 2:1 SR protocol, 35% (38/108) were PLHIV. Subgroup analyses were not pre-specified. For both the 2:1 SR and the Heat + TE methods, sensitivity was significantly lower among PLHIV than among people without HIV (*P* = 0.001 and *P* = 0.003, respectively, two-population proportion *z*-test). There were no significant differences in sensitivity between methods regardless of the patients' HIV status (Fig. 3).

## DISCUSSION

The 2:1 SR method was developed to mitigate two limitations of the Heat + TE method when used to prepare swab samples for Xpert Ultra testing, namely the requirement for a heating instrument and the tendency of Heat + TE to cause overpressure errors in some populations. Here, we used a paired sample comparison to show that the advantages of 2:1 SR come at no cost with regard to the rate of positive detection of TB. In fact, the sensitivity of the 2:1 SR method appeared higher than the Heat + TE method, though the two had overlapping confidence intervals and there was no significant difference between sensitivities. The sensitivities of both methods reported here are artificially low due to the prioritization of samples from low-bacillary load participants. This does not reflect the sensitivity that could be expected if applied to a broader population of symptomatic patients. As has been shown here and in other previous studies, TS positivity is lower among participants with a lower bacillary load, using sputum Xpert Ultra semi-quantitative results as a proxy (5, 7).

High error rates in some populations were reported with the Heat + TE protocol and ranged from 0% to 16% (13). In this smaller study, the overall error rate of the 2:1 SR method was 1.8% (2/110) and the rate of pressure errors was 0.9%. Given that the initial error rate reported for Heat + TE by Wood et al. (7) (1%) was also low, this study could not assess whether the 2:1 SR method reduced the problem. However, *in vitro* experiments with contrived samples, reported by Andama et al. (8) and Chilambi et al. (21), showed that the addition of Cepheid SR to TS samples may reduce error rates in Xpert Ultra. The optimal dilution of SR to buffer was determined to be 2:1 due to higher positive detection rates in samples spiked with smaller cell counts and prior evidence that it is sufficient for sample decontamination.

As has been the case previously (5, 10), the sensitivity of both the Heat + TE method and the 2:1 SR method dropped when used on samples collected from PLHIV. There were also proportionally fewer PLHIV among those participants that were TS true-positive than TS false-negative. PLHIV tend to have lower bacillary load infections than those without HIV (24), which could be the driver of this difference in swab detection. The results of the present study showed that the 2:1 SR method is no more or less sensitive to this quantitative effect than Heat + TE. By either sputum or TS sample analysis, PLHIV remain a population in which effectively diagnosing TB is more challenging.

Xpert Ultra testing of two serially collected samples resulted in only a modest (not statistically significant) increase in sensitivity. Even a modest increase could be beneficial in populations where TS are easier and more manageable to collect than sputum. However, further work using more sensitive, swab-dedicated methods (5, 14) could uncover further improvements in sensitivity with serially collected samples.

This study had several limitations. The sample size was insufficient to fully compare the diagnostic accuracy of the 2:1 SR method relative to Heat + TE. However, in a recent clinical evaluation conducted on 1,168 care-seeking participants in four high-TB burden countries, the new 2:1 SR method performed comparably to prior studies that used Heat + TE, with a low error rate (25). A limitation cited by the authors of that study was the lack of head-to-head comparisons between the two methods, and the current study fills that gap. In the current study, limitations on materials (including Xpert Ultra kits, which are difficult to procure in the United States) prevented us from assessing specificity more fully. Additionally, because two different methods were employed, this study was not able to fully address the utility of serial swabbing as a method of increasing sensitivity. The minor increase in sensitivity reported here may be due to serial swabbing, or it may be due to the difference in method.

Despite these limitations, this study supports the adoption of the 2:1 SR method as the protocol of choice for processing TS for analysis by Xpert Ultra. It has methodological and logistical advantages over the previous Heat + TE method, and no detectable disadvantages with regard to diagnostic accuracy.
